# Optimization of Sugar Extraction Process from Date Waste Using Full Factorial Design Toward Its Use for New Biotechnological Applications

**DOI:** 10.3390/biotech13040039

**Published:** 2024-10-03

**Authors:** Islam Sayah, Mondher Njehi, Nicola Cicero, Vincenzo Nava, Manel Ben M’hadheb, Hatem Majdoub, Sami Achour, Teresa Gervasi

**Affiliations:** 1 Research Unit UR17ES30 “Genomics, Biotechnology and Antiviral Strategies”, Higher Institute of Biotechnology of Monastir, University of Monastir, Tahar Hadded Avenue, PB74, Monastir 5000, Tunisia; sayahislam2011@gmail.com (I.S.); manelbenmhadheb@gmail.com (M.N.); njehimondher@gmail.com (M.B.M.); 2 Department of Biomedical and Dental Sciences and Morphofunctional Imaging, University of Messina, 98168 Messina, Italy; nicola.cicero@unime.it (N.C.); vincenzo.nava@unime.it (V.N.); 3 Laboratory of Interfaces and Advanced Materials, Faculty of Sciences of Monastir, University of Monastir, Monastir 5000, Tunisia; hatem.majdoub@fsm.rnu.tn

**Keywords:** date waste, sugar extraction, optimization, full factorial design

## Abstract

In Tunisia, the date industry generates a large quantity of waste, raising environmental concerns. However, dates are rich in sugars, which offer a renewable source of nutrients for various applications. In this study, sugar extraction from two low-grade pitted date fruits (Alig and Kentichi) under ultrasound, was optimized using full factorial design. At 40 °C, for20 min, and with a liquid-to-solid ratio of 10 mL/g, the optimum sugar contents were 60.87% and 50.79% for the varieties Alig and Kentichi, respectively. The date extracts were chemically analyzed, revealing low fat and protein contents, but significant polyphenol and mineral contents in both varieties. HPLC-IR analysis revealed more inverted sugars (glucose and fructose) in the Alig variety and more sucrose in the Kentichi variety. FTIR and SEM analysis showed the efficiency of the ultrasonic treatment of the biomass in terms of improving mass transfer diffusion through ultrasonic cavitation. Thus, ultrasound-assisted extraction constitutes an effective method for the recovery of sugar from date waste.

## 1. Introduction

In the Middle East and North Africa, dates (*Phoenix dactylifera*) constitute an essential food owing to their richness in valuable nutrients, including carbohydrates (44–88%), dietary fiber (6.4–11.5%), and minerals (such as calcium, magnesium, potassium, and iron), as well as vitamins (B1, B2), fatty acids (0.2–0.5%), and protein (2.3–5.6%) [1]. In 2020, date production exceeded more than 9 million tons worldwide [2]. Tunisia emerged as one of the leading producers of dates, with an annual production estimated at 331,500 tons, primarily represented by the variety Deglet Nour [3].

Despite this progress, the date industry generates a large amount of fruit wastes (25,000 tons in Tunisia) throughout the various stages of the process, including picking, processing, and storage [4]. Typically, date waste is used to feed domestic farm animals or is just discarded into open lands, leading to serious environmental concerns due to the high sugar and water content [5,6]. Prior research has explored the chemical composition and nutritional benefits of date fruits [1,7] as well as their valorization in various fields, particularly in the food industry as a sugar substitute [8,9]. Present investigations focus on the utilization of waste materials and byproducts from different industrial sectors, such as the paper industry, food industry, and bioethanol and biodiesel production, for the production of functional food and fermentation feedstocks [10]. Due to the high amount of soluble sugars (44–88%), many attempts have been made to valorize low-grade date fruits into high-value-added products, including novel food products as well as organic acids, protein, antibiotics, enzymes, biofuels, and biopolymers, through microbial bioconversion [11,12,13].

The conventional extraction process of sugar from date fruits requires a high volume of solvents, a long extraction period, and high operative temperatures. As a result, the obtained product has a dark color and loses some nutritive components. To overcome these challenges, different extraction techniques have been suggested, such as ultrasound-assisted extraction, enzyme-assisted extraction, and microwave-assisted extraction and enzyme-assisted extraction. Among all these techniques, ultrasound-assisted extraction has attracted more attention due to the improvement of solvent penetration and matter transfer [14,15].

The objective of this study is to optimize the sugar extraction from two low-grade pitted date fruits (Alig and Kentichi) assisted by ultrasonic waves using a full factorial design. The optimized extract was subjected to chemical characterization including sugars, proteins, fats, polyphenols, and mineral content. HPLC coupled with Refractive Index Detection (RI) and an ICP-MS ICAP-Q spectrometer were employed to determine the individual sugar profiles and the mineral profiles of the extracts obtained from the two date fruit varieties. FTIR and SEM analyses were conducted to determine the morphological and biochemical characteristics of the dates before and after ultrasonic treatment.

## 2. Materials and Methods

### 2.1. Samples and Reagents

Two varieties of low-grade date fruit (Alig and Kentichi) were purchased from a local market in Nefta, Southern Tunisia. They were washed, pitted, and oven-dried at 45 °C, then ground and sieved to a 0.45 mm particle size.

Solvents and other standards, such as glucose, phenol, sulfuric acid, sodium hydroxide, sodium chloride, boric acid, Folin-Ciocalteu’s phenol reagent, sodium carbonate, gallic acid, Folch reagent, and sodium sulfate, were acquired from Sigma Aldrich (St. Louis, MO, USA).

The standard solutions of sugars including d-(+)-glucose (>99.5%), d-(−)-fructose, (>99%), sucrose (>99.5%), d-(+)-maltose monohydrate (>99%), d-(+)-lactose monohydrate (ACS reagent), and maltotriose (98%) were obtained from Merck, Darmstadt, Germany. Mobile phase constituents included HPLC-grade water and 0.01 N sulfuric acid solution (Fa. Merck, Darmstadt, Germany). 

### 2.2. Ultrasound-Assisted Extraction

Sugar extraction was performed utilizing a bath sonicator (model RK 52 H 164056170, Berlin, Germany) under a stable frequency of 35 kHz and an ultrasonic power set at 12 W. In each experiment, 5 g of date powder (DP) from two different varieties of date fruit was dissolved in a definite volume of distilled water ranging from 6 to 10 mL/g (L/S ratios). The resulting mixture was then treated with ultrasonic waves under the following set conditions: temperature varying between 40 and 75 °C and time ranging from 20 to 60 min.

After each run, the samples were vacuum-filtered to separate the liquid fraction, which contained soluble sugars, from the solid fraction, which was composed of fibers and insoluble components. The liquid extract was analyzed to determine the total sugar content (TSC) using the phenol sulfuric acid method. Meanwhile, the remaining fraction, referred to as date residue, was dried in an oven at 100 °C for 24 h and stored for further analysis as described by ALYammahi et al. [16]. Each experiment was carried out in triplicate.

### 2.3. Full Factorial Experimental Design

The extraction of date sugar using the ultrasound technique is influenced by several operational parameters. In this study, the temperature, extraction time, and liquid-to-solid (L/S) ratio were examined to assess their influence on the TSC and to identify the optimal conditions for achieving maximum sugar concentration in the resulting date extracts. A full factorial design (FFD) with three factors and two levels (−1, +1) was employed to fulfill these objectives. The experimental levels for each variable were chosen according to existing literature. Table 1 and Table 2 provide the experimental matrix developed for the studied date varieties (Alig and Kentichi). The independent variables, represented with both coded and uncoded levels, were as follows: extraction temperature (denoted as X_1_, ranging from 40 °C to 75 °C), extraction time (denoted as X_2_, ranging from 20 to 60 min), and L/S ratio (denoted as X_3_, ranging from 6 to 10 mL per gram). The total sugar content per gram of date powder (TSC mg/g DP), denoted as Y, was used as a response variable and was determined using the phenol–sulfuric acid method. An analysis of variance (ANOVA) was performed with a 95% confidence level to estimate the significance of the independent variables. A statistical model was utilized to identify the optimal conditions for maximizing sugar content, which were then validated through experimentation.

### 2.4. Analysis of Date Extracts

#### 2.4.1. Total Sugar Content

The TSC was determined according to the sulfuric acid and phenol method [17]. The TCS was determined in milligrams of glucose equivalence per g of DP using Equation (1):

(1) $$TSC\;=\frac{Weight\;of\;total\;sugar\;in\;the\;extract}{Weight\;of\;the\;DP}$$

#### 2.4.2. Total Protein Content

The determination of the total protein content was performed using the Kjeldahl method based on the AOAC Official Method 963.15 [18].

#### 2.4.3. Total Fat Content

The total fat content (TFC) was assessed as reported by Folch’s technique. Briefly, 2 g of the sample were homogenized with 20 mL of 2:1 chloroform methanol (*v*/*v*). The mixture was incubated in an ultrasonic bath for 20 min, and then 10 mL of NaCl (0.73%) were added, followed by centrifugation for 20 min at 8000 rpm. The top layer was removed, and the bottom layer was filtrated using sodium sulfate to remove traces of remaining water. The solvent was evaporated using a rotator evaporator, and the lipid extract was air-dried, kept in a desiccator, and weighed until a stable mass was obtained for determining the percentage of fats in the date sample [19].

#### 2.4.4. Total Phenolic Content

The phenolic content (TPC) in the date extracts was determined following the Folin–Ciocalteu method. The total phenol concentration was expressed in milligrams of gallic acid equivalent (mg GAE) per 100 g of dry matter [20].

#### 2.4.5. Individual Sugar Profiles HPLC-IR 

The sugar composition of optimized date extracts was determined with High-Performance Liquid Chromatography coupled with a refractive index detector (HPLC-IR, Shimadzu, Germany) [21].

#### 2.4.6. Determination of Mineral Element Contents 

The mineral content analysis, including magnesium (Mg), potassium (K), sodium (Na), calcium (Ca), iron (Fe), and zinc (Zn), in both date extracts followed the method described by Lo Vecchio et al. [22].

### 2.5. Infrared Spectroscopy FTIR

The infrared (IR) spectra of date fruit (AF, KF), extracts (AL, KL) and solid residues (AR, KR) were determined using a Fourier transform infrared spectrophotometer (BX FTIR system spectrometer, PerkinElmer, MA, USA) covering the range of 4000–500 cm^−1^. The spectrums were constructed using the Origin 6.0 software program [23].

### 2.6. Morphological Characterization Using Scanning Electron Microscopy (SEM)

The surface morphological characteristics of date fruit (AF, KF) and date residue (AR, KR) for the two varieties of dates were examined using Scanning Electron Microscopy (SEM, Apero 2, Thermo Fisher Scientific, Waltham, MA, USA). Samples were fixed on aluminum stubs using double-sided carbon adhesive tape. The analyses were performed under an accelerating voltage of 20 kV with different image magnifications of 50, 100, and 200×.

### 2.7. Statistical Analysis

Minitab software version 19.1 was employed to perform the statistical analysis and the ANOVA test with a 95% confidence level (*p*-values *<* 0.05). All analyses were performed in triplicate, and the results are presented as mean ± standard deviation (SD).

## 3. Results and Discussion

The FFD is a systematic approach used in research and experimentation to examine the effects of multiple factors, each at different levels, and understand their individual and combined effects on a particular response of interest. FFD provides a complete and detailed analysis of how each factor influences the response variable, enabling researchers to make informed decisions [24]. In this study, the effect of three independent variables—temperature, extraction time and L/S ratio, as well as their interactions—on the total sugar contents was investigated using a full factorial design as a DOE. ANOVA was carried out to check the significance of the proposed models and to determine the most significant factors.

### 3.1. Variety Alig

Table 2 shows a coefficient of determination (R^2^ = 0.992) close to 1, which means that 99.26% of the total variations in the data can be explained by the developed model (Equation (2)). The analysis of variance (ANOVA) revealed that the model was statistically significant (*p* < 0.05), confirming its reliable prediction of TSC values. The model F-value of 53.75 further supports the model’s significance, suggesting that its performance was not related to random variation.

**Figure 1:**
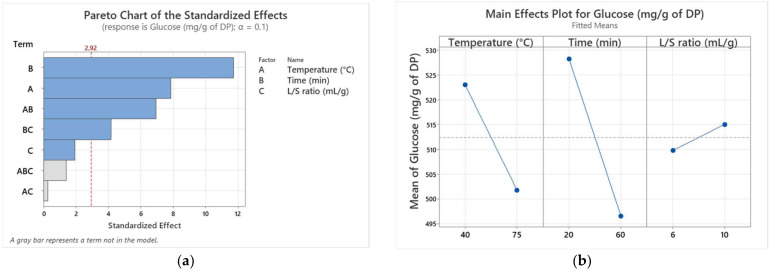
Pareto chart of the standardized effects (**a**); main effects plot for the variety Alig (**b**).

The marginal discrepancy of 1.85% between the R^2^ and R^2^ (adj) values suggests a reduced likelihood of non-significant terms impacting the model. Furthermore, the proximity of the adjusted R^2^ coefficient (0.9741) and predicted R^2^ (0.8818) confirms the strong predictive ability of the developed model. Figure 1a also shows that the effects of first-order factors (temperature, time) and interaction factors (temperature, time) were significant (*p* ≤ 0.05). For additional validation, a Pareto chart was generated, incorporating a reference line (t-value limit) to indicate the significance of each factor, thus ranking them in descending order. Factors crossing this reference line were considered theoretically significant. According to the obtained data, the main effects of time (X_2_), temperature (X_1_), and interaction factors, such as time–temperature (X_2_ × X_1_) and time–L/S ratio (X_2_ × X_3_), exerted a significant influence on the TSC in Alig extract, with the highest coefficients being −15.88, −10.63, −9.37, and −5.62, respectively. On the other hand, the other factors showed no significant effect on the response. Figure 1b illustrates that the extraction time and temperature negatively influenced the TSC in date extract, while the L/S ratio (X_3_) seemed to positively affect the response.

(2) $$Glucose\left(\frac{mg}{g}of\;DP\right)=461.9+0.464\times X1+1.871\times X2+6.94\times X3-0.02679\times X1\times X2-0.1406\times X2\times X3$$

In addition to the main effects, the independent variables (X_1_, X_2_, X_3_) may interact with each other to affect the response (TSC). For instance, extraction time and L/S ratio may interact to affect the TSC in Alig extract. At a short extraction time (20 min), the TSC increases from 519 to 540 mg/g of DP when the L/S ratio increases from 6 to 10 mL/g. However, at a prolonged extraction time (60 min), the TSC decreases from 521 to 512 mg/g of DP (Figure S1 in Supplementary Materials). Therefore, the effect of the L/S ratio varies the response at two different levels of extraction time, suggesting an interaction between these two process parameters. Furthermore, the influence of extraction time on the response (TSC) differs at two different levels of temperature, confirming the significant interaction between these two factors. The TSC reaches its maximum quantity when the temperature and extraction time are both at low levels. Regarding the interaction between temperature and L/S ratio, Figure S1 shows parallel lines, indicating that there is no interaction effect between these factors.

**Figure 2:**
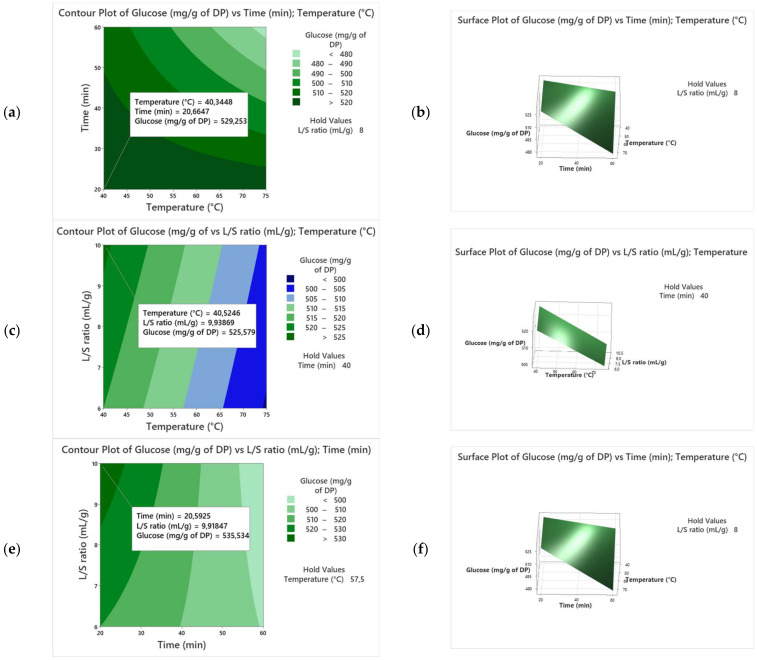
Three-dimensional surface plots and two-dimensional contour plots for the variety Alig. ((**a**,**b**) extraction temperature and time; (**c**,**d**) extraction temperature and liquid-to-solid ratio, and (**e**,**f**) extraction time and liquid-to-solid ratio).

For more details, the 3D surface plots and 2D contour plots (Figure 2) were used to determine the values of the response with different combinations of the independent variables (X_1_, X_2_, X_3_) and to visualize the interactions between these variables. The contour lines or curves link points with constant response values, aiding in the selection of the best combinations that produce the desired response. Linear contour curves suggest the insignificance of the interaction term, while pronounced curvatures in the contours indicate a substantial and crucial interaction term [25]. In our case, the contour curves display significant curvature (Figure 2a), indicating that the interactions X_2_ × X_1_ and X_2_ × X_3_ are significant. However, Figure 2b shows linear lines, which means no significant interaction for X_1_ × X_3_. Furthermore, the contour plot provides the best factor settings for a maximum TSC in the date extract. As shown in our results, the TSC increased from 475 mg/g of DP to 540 mg/g of DP as the extraction temperature and time varied from 40 to 55 °C and 20 to 40 min, respectively, which can be explained by the enhanced solubility of sugar particles [26]. On the other hand, extending the extraction time beyond 40 min led to a rapid reduction in TSC, which can be explained by the degradation of sugar particles and formation of impurities like Maillard reaction products [27].

The extraction process was also conducted by changing the L/S ratios from 6 to10 mL/g while maintaining the time and temperature at 20 min and 40 °C, respectively. As reported in Figure 2c–f, the TSC was enhanced with a variation in the L/S ratio from 6 to 10 mL/g, leading to a decrease in solvent viscosity and dissolution of more sugar molecules in water. Therefore, the maximum TSC was set within the following conditions: 20 min, 10 mL/g, and 40 °C, which were considered for further validation.

**Figure 3:**
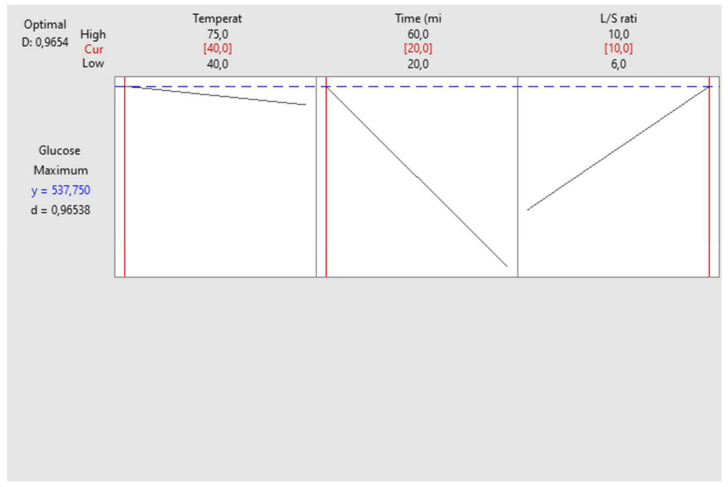
Optimal extraction conditions for Alig variety.

To validate the predicted model, the ultrasound-assisted extraction of date sugar was carried out under the proposed conditions (40 °C, 20 min, and 10 mL/g). The obtained extract yielded a TSC of 608.710 mg glucose/g DP, which closely aligns with the predicted value indicated by the proposed model (537.750 mg glucose eq/g DP), as shown in Figure 3. These results were obtained with a desirability of 96.53%. 

### 3.2. Variety Kentichi

As shown in Table 3, a high coefficient of determination (R^2^ = 99.83%) with a low standard error (1.215) indicates that the model effectively explains the variation in the TSC values in the Kentichi extract. Consequently, the suggested model is expressed by Equation (3).

(3) Glucose(mg/g of DP)=504.45−0.8936×X1−0.684×X2+7.968×X3+0.01565×X1×X2−0.0382×X2×X3

**Figure 4:**
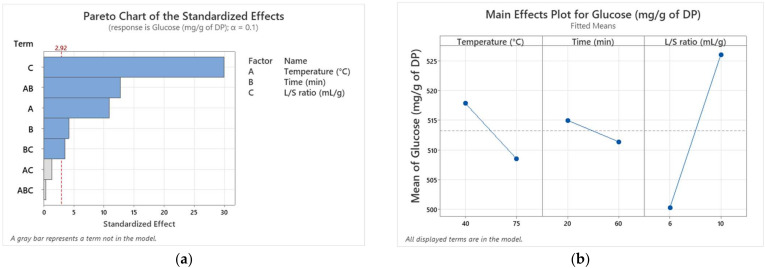
Pareto chart of the standardized effects (**a**); main effects plot for the variety Kentichi (**b**).

The analysis of variance (ANOVA) revealed that the model is highly significant (*p* < 0.01), meaning that it effectively predicts the response values based on the extraction parameters (Table 3). Additionally, the adjusted R^2^ coefficient (0.9736) and predicted R^2^ (0.9942) confirmed a robust correlation between the input variables and the studied response. The effects of first-order (L/S ratio, temperature) and two-way interactions (temperature time) were significant (*p* ≤ 0.01). The Pareto chart (Figure 4a) indicates that the L/S ratio (X_3_), the interaction time temperature (X_2_, X_1_), and the extraction temperature were the most significant factors influencing the TSC in Kentichi extract. Figure 4b reveals the high and positive effect of L/S ratio (X_3_) on TSC in Kentichi extract, while temperature (X_1_) and time (X_2_) exhibited lower negative effects, indicating that increasing the temperature and extraction time leads to a decrease in the extracted sugar. The high effect of the L/S ratio on the response may be explained by the dry texture of the Kentichi variety, which needs more water to dissolve sugar particles and to enhance the matter transfer.

Furthermore, the independent variables (X_1_, X_2_, X_3_) may interact with each other to affect the response (TSC). At a short extraction time (20 min), the TSC decreased from 511.2 to 500 mg/g of DP when the temperature increased from 40 to 75 °C. However, after 60 min of extraction time, the TSC increased from 490 to 500.1 mg/g of DP (Figure S2 in Supplementary Materials). Therefore, the effect of extraction time on the TSC depends on the level of the extraction temperature (min or max), indicating the presence of interaction between these two factors. For the interaction between time and L/S ratio, the results reveal that L/S ratio inversely affects the output at two different extraction times, which confirms the interaction between these two parameters. Parallel lines disprove any interaction between temperature and L/S ratio.

**Figure 5:**
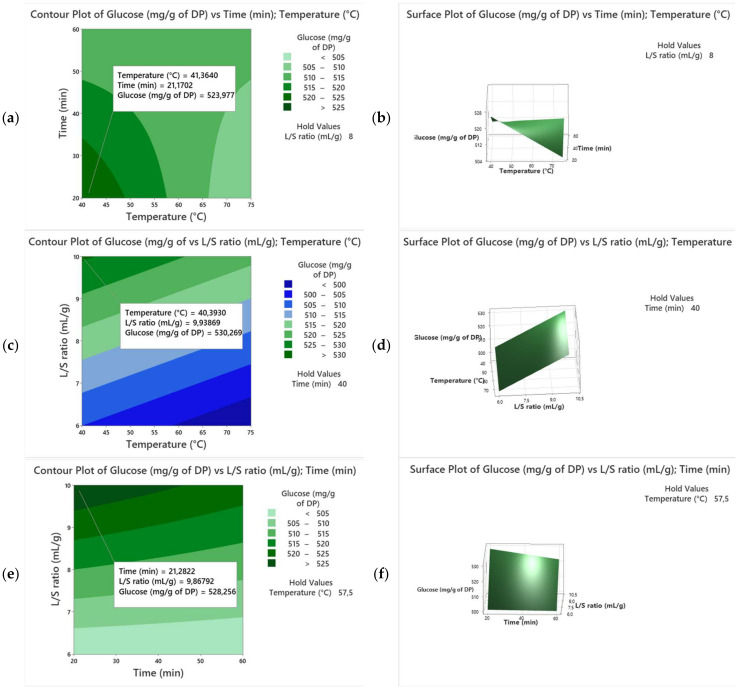
Three-dimensional surface plots and two-dimensional contour plots for the variety Kentichi ((**a**,**b**) temperature versus time; (**c**,**d**) temperature versus liquid-to-solid ratio; and (**e**,**f**) time versus liquid-to-solid ratio).

The 3D surface plots and 2D contour plots (Figure 5) were also utilized to highlight the interactions between the studied variables. Figure 5a,b demonstrate a strong curve, indicating a notable interaction between temperature and time, while Figure 5e,f reveal softer curves, meaning a weaker interaction between time and L/S ratio. The contour plot depicting temperature versus L/S ratio displays linear lines, demonstrating there was no significant interaction between temperature and L/S ratio. The TSC dropped drastically as the extraction temperature and time exceeded 40 °C and 20 min, respectively (Figure 5a,b). The optimal extraction temperature and time resulting in the highest TSC were around 40 °C and 20 min, respectively. In addition, the sugar extraction was also performed by changing the L/S ratios (6 to10 mL/g) while keeping the temperature and extraction time constant. The increase in the L/S ratio from 6 to 10 mL/g ameliorated the TSC in Kentichi extract (Figure 5c–f). Therefore, the maximum TSC was registered under the following conditions: 40 °C, 20 min, and 10 mL/g, which were considered for further validation.

**Figure 6:**
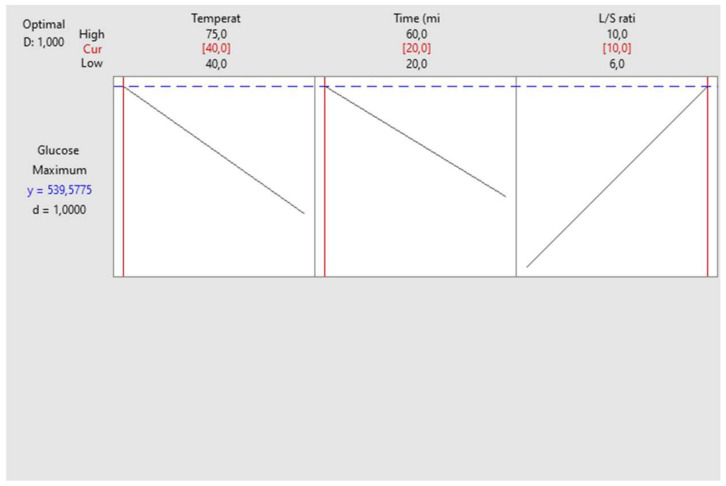
Optimal extraction conditions for Kentichi variety.

To validate the proposed model, the extraction procedure was repeated in triplicate under the optimum conditions described above (40 °C, 20 min, 10 mL/g). The experimental value registered was 507.64 mg glucose/g DP, which is consistent with the predicted value given by the model (539.577 mg glucose eq/g DP), as shown in Figure 6. This result was obtained with a desirability of 100%.

In conclusion, the highest extraction efficiency was achieved for both date varieties under the same following conditions: 40 °C extraction temperature, 20 min extraction time, and 10 mL/g liquid-to-solid ratio. These findings are consistent with previous studies. Alyammahi et al. [16] suggested an extraction temperature of 60 °C, an extraction time of 30 min, and an L/S ratio of 7.6 mL/g to obtain the optimum sugar content from the Sukkari date variety. According to Entezari et al. [28], a low temperature (15 °C) and a high water/fruit ratio (9/1) and ultrasonic intensity provide the best sugar content from date fruit. Lin et al. [29], who studied the extraction of polysaccharide from *Ziziphus jujube* Mill var *spinosa* seeds (ZSS) using ultrasound-assisted extraction (UAE), confirmed the significant effects of extraction time and sonication power on extraction yield. They reported the following optimizing conditions: 52.5 °C, 21.2 min, 134.9 W, and L/S ratio of 26.3 mL/g. In our study, a maximum sugar content was obtained using less energy (only 40 °C) and a short extraction time (20 min), leading to a reduction in the processing cost compared to conventional extraction methods.

### 3.3. Characterization of Optimized Date Extracts

After optimizing the sugar extraction process, date extracts of the studied varieties were assessed for their sugar and mineral profiles and their composition in terms of protein, fats, and polyphenols. The results are summarized in Table 4.

Sugars were the most abundant component in both varieties, ranging from 50.79% to 60.2% in Kentichi and Alig, respectively. Alig extract showed a higher sugar percentage than Kentichi extract, with a *p* < 0.001. Our results are lower compared to those obtained by AlYammahi et al. [16], with 812 mg/g of DP for the Sukkari variety, and those provided by Messadi et al. [9], with 679.1 mg/g of DP for the Kentichi variety. The variations in sugar content are attributed to the fruit variety and grade. In fact, in the present study, we used a very low quality of date fruit which is usually considered as waste. The protein content was quite low for both varieties, with values of 1.39 g/l and 1.07 g/l for Alig and Kentichi, respectively, with a significant difference between varieties (*p* < 0.001). A negligible amount of fat was registered, with values of 0.001% and 0.002% for the varieties Alig and Kentichi, respectively. Furthermore, a good level of polyphenols was found, with amounts of 333 mg eq GAE/100g and 254 mg eq GAE/100g for the Alig and Kentichi varieties, respectively. These amounts are lower than those provided by Saafi et al. [30] and higher than those reported by Lajnef et al. [31], who found a total phenolic content of 138.97 mg eq GAE/100 g in date syrup. The differences observed in the nutritional composition of both varieties of dates when compared to the literature could be explained by several factors, such as the variety, date cultivar, geographic origin, harvest season, solvent used, and extraction method (process parameters) [32]. 

For a more detailed view, the sugar profiles of date extracts were screened via high-performance liquid chromatography with refraction index detection, and the results are displayed in Table 5. They showed that the amount of reducing sugars was significantly higher in Kentichi extract than in Alig extract (*p* < 0.001). Moreover, the composition of simple sugars revealed some differences between the studied varieties of dates. For example, in the Alig extract, fructose (35.4%) and glucose (30.3%) were the predominant sugars, with the sucrose content being minimal (0.2%). However, the sucrose content was significantly higher in the Kentichi extract (22.8%) compared to Alig. Additionally, other simple sugars were present, including maltose, maltotriose, and lactose, with amounts of 3.8%. 2.1%, and 1%, respectively. Good levels of fructose (24%) and glucose (17.9%) were also registered in the Kentichi extract. These differences in the sugar composition may be related to the fruit texture. Typically, soft date varieties are rich in reducing sugars (glucose and fructose), with a low amount of sucrose, whereas dry date varieties contain much higher contents of sucrose [33]. These results are consistent with the value given by AlYammahi et al. [16] in their study of a common date variety (Degla-beida). They reported a high sucrose concentration in the Sukkari date variety (~85 mg/mL). In another study, Messadi et al. [9] indicated the predominance of sucrose (72%) over glucose (14.26%) and fructose (13.74%) in Kentichi extract. Notably, maltose and lactose were no longer present in Kentichi extract after 2 h of extraction time. In our case, the presence of maltose, maltotriose, and lactose may be explained by the short extraction time (20 min) employed. These findings demonstrate that high temperature and longer extraction time alter the sugar composition of dates.

Date fruits are further recognized for their high content of minerals. Concentrations of essential minerals such as K, Mg, Ca, Na, Fe, and Zn for the studied date extracts were examined by using an ICP-MS iCAP-Q spectrometer and are provided in Table 6. The results show that potassium (K) was the major mineral element present in both date extracts, with values of 363.79 mg/100 g of DP and 349.82 mg/100 g of DP, respectively, for the varieties Alig and Kentichi. The macroelement profile reveals high Mg, Ca, and Na concentrations in the ranges of 40.82–43.01 mg/100 g of DP, 38.02–38.32 mg/100 g of DP, and 9.60–9.86 mg/100 g of DP, respectively, for both varieties of date fruit. Iron and zinc essential microelements were found in high amounts ranging between 1.56–1.67 mg/100 g of DP and 0.95–1.09 mg/100 g of DP, respectively, for the studied varieties. These results are consistent with the values presented by Tang et al. [32], who studied the chemical compositions of various date fruit varieties.

Date extract provides several benefits due to its rich nutritional profile and antioxidant content, and it could be a better option than commercially refined sugar. Several studies have shown the ability to use date sugar extract in many sectors, particularly in the food industry. A study carried out by Lajnef et al. [31] reported the use of date syrup as a sugar replacement in sponge cake. Previous results have shown that the final product has a texture profile which is very close to the control, with high antioxidant activity. Moreover, Rangaraj et al. [34] demonstrated that the incorporation of date fruit syrup waste extract in gelatin films delays lipid peroxidation and extends the shelf life of canola oil. AlYammahi et al. [35] developed a fortified camel milk powder enriched with date sugar extract using the spray-drying technique. The final product has a high nutritional value, with a high content of poly- and mono-unsaturated fats, a low cholesterol content, and good thermal stability, making it a value-added food. Furthermore, date extract could be used as a potential substrate for microbial fermentations. Salah-Tazdaït et al. [36] examined the use of crude juice from dates as an additional carbon source for malathion degradation by *Stenotrophomonas maltophilia*. Previous results have indicated a significant increase in biomass growth, leading to the improvement of malathion biodegradability. Also, Elsanhoty et al. [37] investigated the production of carotenoids by *Lactobacillus plantarum QS3* using date syrup as a source of sugar. The obtained results indicated that 5% of date syrup produces 16.21 mg/kg dry cell of carotenoids. A similar study conducted by Acourene and Ammouche. [38] demonstrated that date waste is a potential substrate for the synthesis of several fermentation products, including ethanol, citric acid, and α-amylase. Thus, we have observed that agro-industrial by-products such as date waste may serve as great ingredients for various biotechnological applications. Furthermore, date extract was successfully used as a carbon source to enhance biopolymer production yield. For example, Lotfiman et al. [39] revealed a 68% increase in bacterial cellulose production yield using 3% (*w*/*v*) dates in the medium after 8 days of incubation compared to the standard medium. 

Therefore, striving to expand the circular economy concept helps to ensure a safe environment and a high quality of life for humankind.

### 3.4. FT-IR Analysis

**Figure 7:**
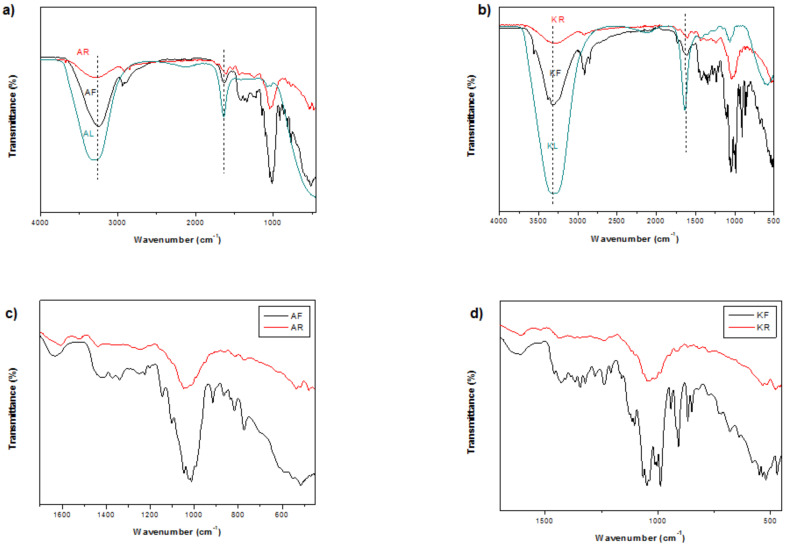
FT-IR spectra of Alig fruit (AF), Alig residue (AR), and Alig liquid extract (AL) (**a**,**b**), as well as Kentichi fruit (KF), Kentichi residue (KR), and Kentichi liquid extract (KL) (**c**,**d**).

The FTIR spectra of date fruit, solid residue, and liquid extract for the studied date varieties are presented in Figure 7. The date fruit spectrum (AF, KF) showed a large band at 3255 cm^−1^ attributed to hydroxyl groups (–OH). The characteristic band at 2942 cm^−1^ corresponded to the CHO group. The typical absorption band at 1640 cm^−1^ was associated with the vibration of C=C aromatic and COO^–^. Several bands were detected at the fingerprint region between 1500 and 450 cm^−1^, corresponding to the carboxylic group (C=O) of carbohydrates, acids, ketones, and aldehydes. A strong absorbance was registered around 1170 to 1050 cm^−1^, which was attributed to polysaccharides. However, in the date residue spectrum, no significant peaks were detected for either variety of date, indicating that sugar molecules and other phytochemicals were successfully extracted from the date fruit. Additionally, the spectral pattern did not change after sonication, indicating that the chemical composition of the studied material was not altered. This suggests that sonication treatment is a potential tool for molecule recuperation with less damage. Therefore, ultrasound extraction demonstrated a strong performance in terms of extracting sugar molecules from date fruits while preserving the chemical integrity. These results are in line with those provided by AlYammahi et al. [16], who performed sugar extraction using another variety of dates.

### 3.5. Scanning Electron Microscopy (SEM)

**Figure 8:**
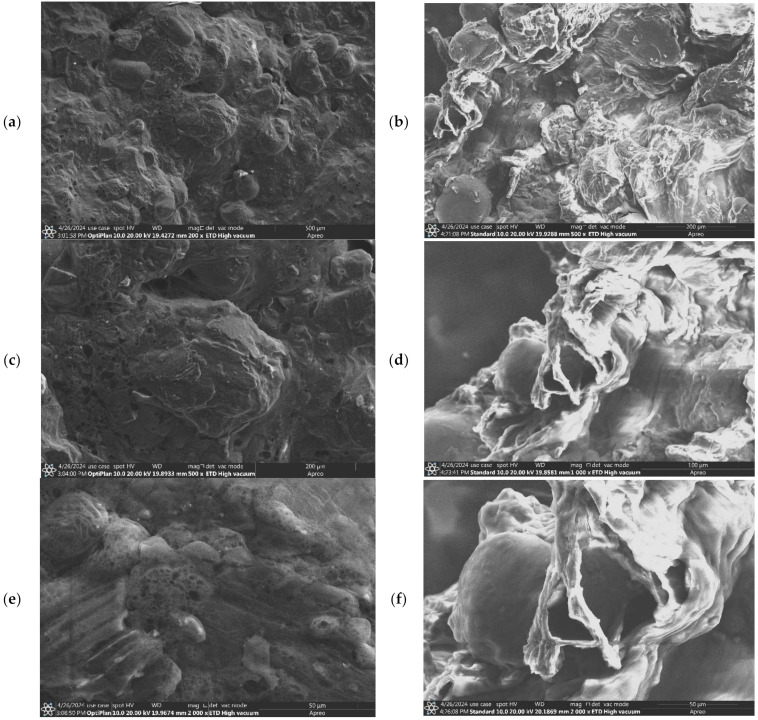
SEM images with different magnifications of Alig fruit (AF) (**a**,**c**,**e**), Alig residue (AR) (**b**,**d**,**f**).

**Figure 9:**
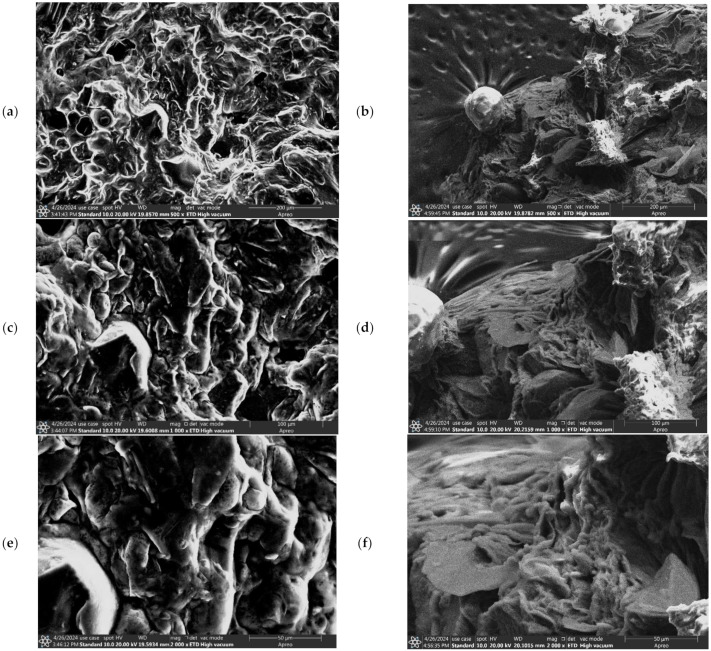
SEM images with different magnifications of Kentichi fruit (KF) (**a**,**c**,**e**), and Kentichi residue (KR) (**b**,**d**,**f**).

The surface morphology of Alig fruit (AF), Kentichi fruit (KF), Alig residue (AR), and Kentichi residue (KR) was assessed using SEM and is displayed in Figure 8 and Figure 9. The untreated date samples (AF, KF) showed firm and compact surfaces (Figure 8a,c,e and Figure 9a,c,e). However, much damage was recorded with the treated ones (AR, KR) using different magnifications. As shown in Figure 8b,d,f and Figure 9b,d,f, many holes and cracks with sharp edges were generated on the surfaces of both varieties of date. These surface changes are explained by the strong effect of the acoustic cavitation generated by the ultrasonic wave, which attacked the cell walls and released the internal content [40]. In fact, the ultrasound waves navigated through the liquid solvent while producing acoustic cavitation, which caused particle fragmentation, cell disruption, and sonoporation. Therefore, the permeability of cell walls was improved, and the extraction yield was boosted [41]. Our results were in agreement with those provided by Santos et al. [42] and Dias et al. [43], who showed that the use of ultrasound waves for vegetal materials improved the extraction yield due to the lateral cracks formed, which led to the release of internal particles.

In conclusion, the ultrasound extraction technique represents a promising alternative to conventional methods for the isolation of valuable nutrients from vegetal matrices [44].

## 4. Conclusions

In this study, sugar extraction from date waste was successfully optimized using full factorial design. The highest sugar contents for both varieties reached 60.87% and 50.79% for the Alig and Kentichi varieties, respectively, under the following optimal extraction conditions: a lower temperature of 40 °C, a shorter extraction time of 20 min, and an L/S ratio of 10 mL/g, leading to less energy consumption. Optimized date extracts revealed strong potential in terms of carbohydrates, polyphenols, and minerals, and they may serve as a potential ingredient in several biotechnological applications, such as functional food, biofuels, organic acids, and production of biopolymers.

In our research, the ultrasound-assisted extraction technique yielded excellent results in terms of improving sugar extraction while preserving the nutritional properties (minerals, polyphenols) of the resulting extracts.

Adopting the concept of circular economy, turning agricultural waste and byproduct streams into valuable products not only minimizes environmental impact, but also unlocks new and significant economic opportunities, fostering innovation and sustainability in various industries.

## Figures and Tables

**Table 1:** FFD experimental matrix of ultrasonic-assisted extraction of sugars from Alig and Kentichi date varieties.

Runs	X_1_	X_2_	X_3_	Y (Alig)	Y (Kentichi)
1	75 (+1)	20 (−1)	10 (+1)	533	519.69
2	40(−1)	60 (+1)	10 (+1)	512	521.22
3	75 (+1)	60 (+1)	6 (−1)	478	500.1
4	75 (+1)	20 (−1)	6 (−1)	521	490
5	40 (−1)	20 (−1)	6 (−1)	519	511.2
6	40 (−1)	20 (−1)	10 (+1)	540	539.14
7	40 (−1)	60 (+1)	6 (−1)	521	500
8	75 (+1)	60 (+1)	10 (+1)	475	524.28

X1: temperature (°C), X2: time (min), X3: L/S ratio (mL/g), Y: glucose (mg/g of DP).

**Table 2:** Regression analysis (ANOVA) of full factorial design for Alig variety.

Source	DF	Adj SS	Adj MS	*F*-Value	*p*-Value
Model	5	3930.63	786.13	53.75	**0.018**
Linear	3	2974.37	991.46	67.79	0.015
X_1_	1	903.13	903.13	61.75	0.016
X_2_	1	2016.12	2016.12	137.85	0.007
X_3_	1	55.13	55.13	3.77	0.192
2-Way Interactions	2	956.25	478.12	32.69	0.030
X_1_ × X_2_	1	703.12	703.12	48.08	0.020
X_2_ × X_3_	1	253.12	253.12	17.31	0.053
Error	2	29.25	14.63		
Total	7	3959.88			

S = 3.82426, *R^2^* = 99.26%, *R^2^* (pred) = 88.18%, *R^2^* (adj) = 97.41%. X_1_: temperature (°C), X_2_: time (min), X_3_: L/S ratio (mL/g).

**Table 3:** Regression analysis (ANOVA) of full factorial design for Kentichi variety.

Source	DF	Adj SS	Adj MS	*F*-Value	*p*-Value
**Model**	5	1787.22	357.44	241.84	**0.004**
**Linear**	3	1528.61	509.54	344.75	0.003
**X_1_**	1	175.69	175.69	118.87	0.008
**X_2_**	1	26.03	26.03	17.61	0.052
**X_3_**	1	1326.90	1326.90	897.76	0.001
**2-Way Interactions**	2	258.61	129.31	87.49	0.011
**X_1_** ** × ** **X_2_**	1	239.91	239.91	162.32	0.006
**X_2_** ** × ** **X_3_**	1	18.70	18.70	12.65	0.071
**Error**	2	2.96	1.48		
**Total**	7	1790.18			

S = 1.21574, R^2^ = 99.83%. R^2^ (pred) = 99.42%R^2^ (adj) = 97.36%. X_1_: temperature (°C), X_2_: time (min), X_3_: L/S ratio (mL/g).

**Table 4:** Nutritional composition of date extracts of two varieties of date fruit.

Parameter	Alig Variety	Kentichi Variety	*p*-Value
Sugar (%)	60.2 ± 20.35	50.7 ± 4.617	0.001
Protein (%)	0.13 ± 0.004	0.1 ± 0.003	0.001
Fats (%)	0.01 ± 0.0023	0.01 ± 0.0104	0.139
Mineral (%)	0.46 ± 0.02	0.44 ± 0.01	0.39
TPC (mg eq GAE/100 g)	333 ± 4.16	254 ± 6.08	<0.001

Results are expressed as mean values ± standard deviation (*n* = 3).

**Table 5:** Sugar profiles of two date varieties.

Parameter (%)	Kentichi Variety	Alig Variety	*p*-Value
Glucose	17.9 ± 2	30.3 ± 1.2	0.01
Fructose	24 ± 1.3	35.4 ± 0.8	0.001
Sucrose	22.8 ± 0.9	0.2 ± 0.18	<0.001
Maltose	3.8	0	
Lactose	1	0	
Maltotriose	2.1	0	
Total	71.6	65.9	<0.0001

Results are expressed as mean values ± standard deviation (*n* = 3).

**Table 6:** Mineral profiles of date varieties.

Mineral Element (mg/100 g of DP)	Alig Variety	Kentichi Variety	*p*-Value
Fe	1.56 ± 0.1	1.67 ± 0.125	0.29
Na	9.61 ± 0.29	9.86 ± 0.11	0.235
Ca	38.32 ± 1	38.01 ± 1.01	0.725
Mg	40.82 ± 1.21	43.01 ± 0.41	0.041
K	363.79 ± 3.39	349.82 ± 7.61	0.044
Zn	1.09 ± 0.07	0.95 ± 0.11	0.136

Results are expressed as mean values ± standard deviation (*n* = 3).

## Data Availability

The original contributions presented in the study are included in the article/Supplementary Materials, further inquiries can be directed to the corresponding authors.

## Supplementary Materials

The following supporting information can be downloaded at: https://www.mdpi.com/article/10.3390/biotech13040039/s1, Figure S1: Interaction effects of Temperature (X1), time (X2), L/S ratio (X3) for the variety Alig; Figure S2: The interaction plot for the variety Kentichi.

## References

[1] Al-Shahib W., Marshall R.J. (2003). The Fruit of the Date Palm: Its Possible Use as the Best Food for the Future?. Int. J. Food Sci. Nutr..

[2] Boughzala Y., Ben Mahmoud N. (2022). Valorisation de La Filière Dattes En Tunisie Par l’association Savoirs et Techniques “Modernes” et “Traditionnels”: Difficultés, Succès et Perspectives. Sci. Technol. Développement.

[3] General Directorate of Agricultural Studies and Development Agricultural Statistics Yearbook 2020, Tunisia. http://www.onagri.nat.tn/uploads/statistiques/Annuaire-Statistique-2020VF.pdf.

[4] Smaali I., Jazzar S., Soussi A., Muzard M., Aubry N., Nejib Marzouki M. (2012). Enzymatic Synthesis of Fructooligosaccharides from Date By-Products Using an Immobilized Crude Enzyme Preparation of β-Dfructofuranosidase from Aspergillus Awamori NBRC 4033. Biotechnol. Bioprocess Eng..

[5] Al-Farsi M.A., Lee C.Y. (2008). Nutritional and Functional Properties of Dates: A Review. Crit. Rev. Food Sci. Nutr..

[6] Ashraf Z., Hamidi-Esfahani Z. (2011). Date and Date Processing: A Review. Food Rev. Int..

[7] Chaira N., Smaali M.I., Martinez-Tomé M., Mrabet A., Murcia M.A., Ferchichi A. (2009). Simple Phenolic Composition, Flavonoid Contents and Antioxidant Capacities in Water-Methanol Extracts of Tunisian Common Date Cultivars (*Phoenixdactylifera* L.). Int. J. Food Sci. Nutr..

[8] El-Sharnouby G.A., Al-Eid S.M., Al M.M. (2009). Utilization of Enzymes in the Production of Liquid Sugar from Dates. Afr. J. Biochem. Res..

[9] Messadi N., Mechmeche M., Setti K., Tizemmour Z., Hamdi M., Kachouri F. (2023). Optimization of Extraction Parameters and Characterization of Tunisian Date Extract: A Scientific Approach Toward Their Utilization. Sugar Tech.

[10] Koutinas A.A., Vlysidis A., Pleissner D., Kopsahelis N., Lopez Garcia I., Kookos I.K., Papanikolaou S., Kwan T.H., Lin C.S.K. (2014). Valorization of Industrial Waste and By-Product Streams via Fermentation for the Production of Chemicals and Biopolymers. Chem. Soc. Rev..

[11] Nazari S.H. (2011). Sonicated Date Syrup Media Preparation for Microbial Culture. Afr. J. Biotechnol..

[12] Chandrasekaran M., Bahkali A.H. (2013). Valorization of Date Palm (*Phoenix dactylifera*) Fruit Processing by-Products and Wastes Using Bioprocess Technology—Review. Saudi J. Biol. Sci..

[13] Ahmad A., Naqvi S.A., Jaskani M.J., Waseem M., Ali E., Khan I.A., Faisal Manzoor M., Siddeeg A., Aadil R.M. (2021). Efficient Utilization of Date Palm Waste for the Bioethanol Production through Saccharomyces Cerevisiae Strain. Food Sci. Nutr..

[14] Ojha K.S., Aznar R., O’Donnell C., Tiwari B.K. (2020). Ultrasound Technology for the Extraction of Biologically Active Molecules from Plant, Animal and Marine Sources. TrAC Trends Anal. Chem..

[15] Watrelot A.A., Bouska L. (2022). Optimization of the Ultrasound-Assisted Extraction of Polyphenols from Aronia and Grapes. Food Chem..

[16] AlYammahi J., Hai A., Krishnamoorthy R., Arumugham T., Hasan S.W., Banat F. (2022). Ultrasound-Assisted Extraction of Highly Nutritious Date Sugar from Date Palm (*Phoenix dactylifera*) Fruit Powder: Parametric Optimization and Kinetic Modeling. Ultrason. Sonochem..

[17] Dubois M., Gilles K.A., Hamilton J.K., Rebers P.A., Smith F. (1956). Colorimetric Method for Determination of Sugars and Related Substances. Anal. Chem..

[18] (2023). Official Methods of Analysis, 22nd Edition. https://www.aoac.org/official-methods-of-analysis/.

[19] Washburn K.W. (1989). A Modification of the Folch Method of Lipid Extraction for Poultry. Poult. Sci..

[20] Cicco N., Lanorte M.T., Paraggio M., Viggiano M., Lattanzio V. (2009). A Reproducible, Rapid and Inexpensive Folin-Ciocalteu Micro-Method in Determining Phenolics of Plant Methanol Extracts. Microchem. J..

[21] Muntean E., Bărăscu N. (2023). Soluble Carbohydrates in Several Transylvanian Potato Cultivars. Plants.

[22] Lo Vecchio G., Cicero N., Nava V., Macrì A., Gervasi C., Capparucci F., Sciortino M., Avellone G., Benameur Q., Santini A. (2022). Chemical Characterization, Antibacterial Activity, and Embryo Acute Toxicity of *Rhus Coriaria* L. Genotype from Sicily (Italy). Foods.

[23] Mzoughi Z., Souid G., Timoumi R., Le Cerf D., Majdoub H. (2019). Partial Characterization of the Edible Spinacia Oleracea Polysaccharides: Cytoprotective and Antioxidant Potentials against Cd Induced Toxicity in HCT116 and HEK293 Cells. Int. J. Biol. Macromol..

[24] Jankovic A., Chaudhary G., Goia F. (2021). Designing the Design of Experiments (DOE)—An Investigation on the Influence of Different Factorial Designs on the Characterization of Complex Systems. Energy Build..

[25] National Institute of Standards and Technology US Department of Commerce. https://www.itl.nist.gov/Div898/Handbook/Eda/Section3/Eda33a1.Htm.

[26] Chen C., Zhang B., Huang Q., Fu X., Liu R.H. (2017). Microwave-Assisted Extraction of Polysaccharides from Moringa Oleifera Lam. Leaves: Characterization and Hypoglycemic Activity. Ind. Crops Prod..

[27] Maran J.P., Sivakumar V., Thirugnanasambandham K., Sridhar R. (2013). Optimization of Microwave Assisted Extraction of Pectin from Orange Peel. Carbohydr. Polym..

[28] Entezari M.H., Nazari S.H., Haddad Khodaparast M.H. (2004). The Direct Effect of Ultrasound on the Extraction of Date Syrup and Its Micro-Organisms. Ultrason. Sonochem..

[29] Lin T., Liu Y., Lai C., Yang T., Xie J., Zhang Y. (2018). The Effect of Ultrasound Assisted Extraction on Structural Composition, Antioxidant Activity and Immunoregulation of Polysaccharides from Ziziphus Jujuba Mill Var. Spinosa Seeds. Ind. Crops Prod..

[30] Saafi E.B., El Arem A., Chahdoura H., Flamini G., Lachheb B., Ferchichi A., Hammami M., Achour L. (2022). Nutritional Properties, Aromatic Compounds and in Vitro Antioxidant Activity of Ten Date Palm Fruit (*Phoenix dactylifera* L.) Varieties Grown in Tunisia. Braz. J. Pharm. Sci..

[31] Lajnef I., Khemiri S., Ben Yahmed N., Chouaibi M., Smaali I. (2021). Straightforward Extraction of Date Palm Syrup from *Phoenix dactylifera* L. Byproducts: Application as Sucrose Substitute in Sponge Cake Formulation. J. Food Meas. Charact..

[32] Tang Z., Shi L., Aleid S.M. (2013). Date Fruit: Chemical Composition, Nutritional and Medicinal Values, Products. J. Sci. Food Agric..

[33] Ghnimi S., Umer S., Karim A., Kamal-Eldin A. (2017). Date Fruit (*Phoenix dactylifera* L.): An Underutilized Food Seeking Industrial Valorization. NFS J..

[34] Rangaraj V.M., Rambabu K., Banat F., Mittal V. (2021). Effect of Date Fruit Waste Extract as an Antioxidant Additive on the Properties of Active Gelatin Films. Food Chem..

[35] AlYammahi J., Rambabu K., Thanigaivelan A., Hasan S.W., Taher H., Show P.L., Banat F. (2023). Production and Characterization of Camel Milk Powder Enriched with Date Extract. LWT.

[36] Salah-Tazdaït R., Tazdaït D., Berrahma R., Abdi N., Grib H., Mameri N. (2018). Isolation and Characterization of Bacterial Strains Capable of Growing on Malathion and Fenitrothion and the Use of Date Syrup as an Additional Substrate. Int. J. Environ. Stud..

[37] Elsanhoty R.M., Al-Turki I.A., Ramadan M.F. (2012). Screening of Medium Components by Plackett–Burman Design for Carotenoid Production Using Date (*Phoenix dactylifera*) Wastes. Ind. Crops Prod..

[38] Acourene S., Ammouche A. (2012). Optimization of Ethanol, Citric Acid, and α-Amylase Production from Date Wastes by Strains of Saccharomyces Cerevisiae, Aspergillus Niger, and Candida Guilliermondii. J. Ind. Microbiol. Biotechnol..

[39] Lotfiman S., Awang Biak D.R., Ti T.B., Kamarudin S., Nikbin S. (2018). Influence of Date Syrup as a Carbon Source on Bacterial Cellulose Production by *Acetobacter Xylinum* 0416. Adv. Polym. Technol..

[40] Xie P.J., Huang L.X., Zhang C.H., You F., Zhang Y.L. (2015). Reduced Pressure Extraction of Oleuropein from Olive Leaves (*Olea Europaea* L.) with Ultrasound Assistance. Food Bioprod. Process..

[41] Ferarsa S., Zhang W., Moulai-Mostefa N., Ding L., Jaffrin M.Y., Grimi N. (2018). Recovery of Anthocyanins and Other Phenolic Compounds from Purple Eggplant Peels and Pulps Using Ultrasonic-Assisted Extraction. Food Bioprod. Process..

[42] Santos P., Aguiar A.C., Barbero G.F., Rezende C.A., Martínez J. (2015). Supercritical Carbon Dioxide Extraction of Capsaicinoids from Malagueta Pepper (*Capsicum Frutescens* L.) Assisted by Ultrasound. Ultrason. Sonochem..

[43] Dias A.L.B., Arroio Sergio C.S., Santos P., Barbero G.F., Rezende C.A., Martínez J. (2016). Effect of Ultrasound on the Supercritical CO2 Extraction of Bioactive Compounds from Dedo de Moça Pepper (*Capsicum Baccatum* L. Var. Pendulum). Ultrason. Sonochem..

[44] Dong W., Chen Q., Wei C., Hu R., Long Y., Zong Y., Chu Z. (2021). Comparison of the Effect of Extraction Methods on the Quality of Green Coffee Oil from Arabica Coffee Beans: Lipid Yield, Fatty Acid Composition, Bioactive Components, and Antioxidant Activity. Ultrason. Sonochem..

