# Supplementary material for: Optimization of Sugar Extraction Process from Date Waste Using Full Factorial Design Toward Its Use for New Biotechnological Applications
Source: BioTech (Basel). 2024 Oct 3;13(4):39. doi: 10.3390/biotech13040039 (PMC11503435; doi:10.3390/biotech13040039)
Supplement: Supplementary file 1 [file biotech-13-00039-s001.zip › biotech-3197848-supplementary.pdf]

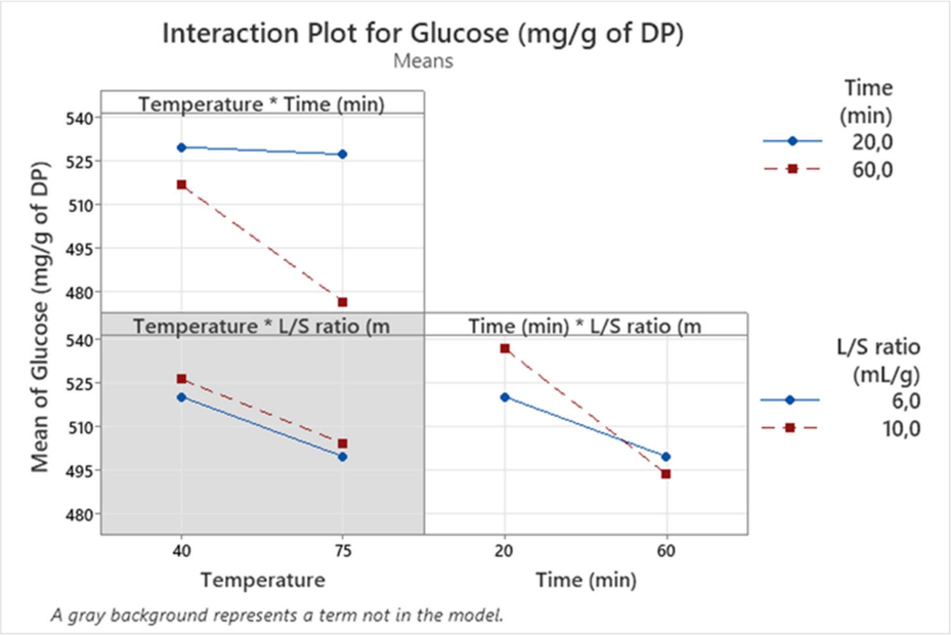


**Figure S1. Interaction effects of Temperature (X1), time (X2), L/S ratio (X3) for the variety Alig**

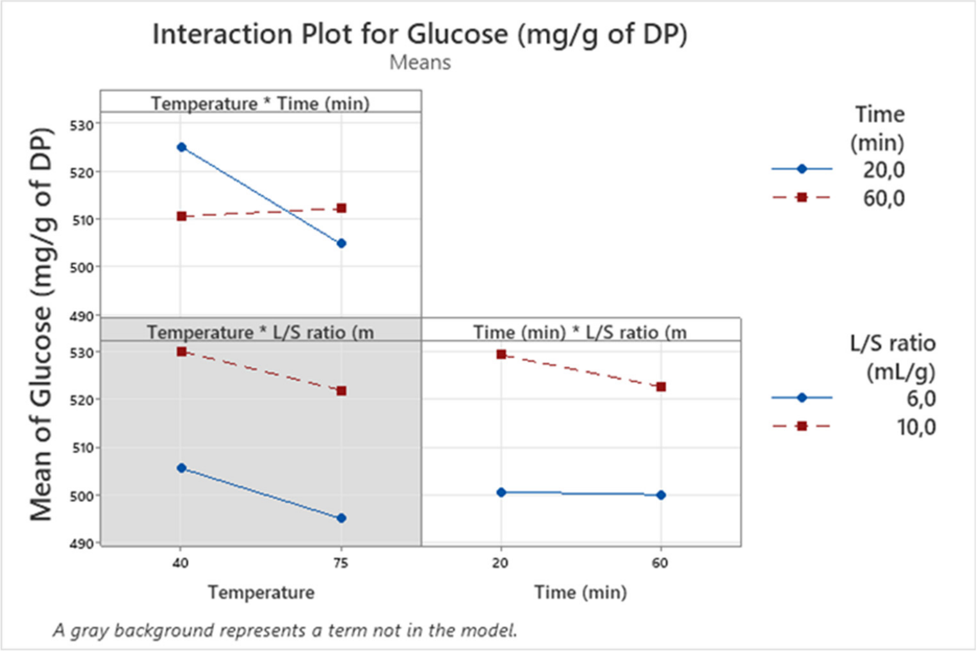


**Figure S2. The interaction plot for the variety Kentichi**
